# A Puzzling Pair: Flail Leg Syndrome with Myokymia and Avascular Hip Necrosis—Case Study and Systematic Literature Review

**DOI:** 10.3390/jcm14196955

**Published:** 2025-10-01

**Authors:** Timotej Petrijan, Marija Menih, Saša Gselman

**Affiliations:** University Department of Neurology, University Medical Centre Maribor, 2000 Maribor, Slovenia; marija.menih@gmail.com (M.M.); sasa.gselman@gmail.com (S.G.)

**Keywords:** radiation therapy, lower motor neuron syndrome, myokymia, osteoradionecrosis, cervical cancer

## Abstract

**Background**: Radiation-induced lower motor neuron syndrome (LMNS) represents a rare but significant delayed complication of oncologic treatment. **Methods**: We present the case of a 56-year-old female who developed LMNS, myokymia, and osteoradionecrosis of the hip nearly two decades after receiving pelvic radiation therapy for cervical carcinoma. To the best of our knowledge, no previous reports have described this particular triad of neurological and tissue changes following radiation therapy. This clinical presentation is analyzed within the framework of a systematic review encompassing 58 documented cases, including our own. **Results**: The database searches yielded 530 records. In total, 32 studies were included in the qualitative synthesis, reporting 57 unique cases of post-radiation LMNS. With the addition of our present case, the final analysis comprised 58 cases. The majority of analyzed patients were male (77.2%), and the most frequent primary malignancies were germ cell tumors (57.9%). The mean age of the analyzed patients at symptom onset was 40.5 ± 13 years, with radiotherapy administered at a mean age of 30.3 ± 12.5 years. The latency period between radiation exposure and the emergence of neurological symptoms averaged 10.2 ± 8.7 years. The mean cumulative radiation dose was 49.0 ± 14.0 Gy. Myokymic discharges were identified in 6 patients (10.3% of cases). Comparative analysis revealed no significant clinical or radiological differences across malignancy subtypes in the manifestation of post-radiation LMNS. **Conclusions**: These findings highlight the need for long-term surveillance of irradiated patients.

## 1. Introduction

Radiation therapy remains a cornerstone in the management of neoplasms, owing to its therapeutic efficacy and generally favourable tolerability profile. Nevertheless, its use is not without risk, as ionizing radiation can inflict collateral damage on adjacent healthy tissues, resulting in a spectrum of complications ranging from mild functional impairment to severe, irreversible injury. A central mechanism of radiation-induced injury involves DNA damage, which, when inadequately repaired, can culminate in apoptotic cell death or contribute to late-onset sequelae such as secondary malignancies. The pathophysiology of radiation-induced neural injury is biphasic: an initial phase characterized by direct cellular damage and acute inflammation, followed by a chronic phase marked by microvascular injury—specifically to small arterioles—and progressive perineural fibrosis, ultimately leading to substantial and often irreversible neural dysfunction. The spinal cord is particularly susceptible, with recognized complications including both early and delayed radiation myelopathy, lower motor neuron syndrome (LMNS), and, in rare instances, spinal hemorrhage [1].

Lower motor neuron syndrome (LMNS), a condition that clinically mimics motor neuron disease, may develop as a delayed complication of radiation therapy involving the spinal cord. It is most frequently observed in patients treated for testicular cancer or lymphoma. The syndrome typically presents with progressive lower limb weakness, muscle fasciculations, and atrophy, while sensory modalities remain preserved. In some cases, however, patients may also exhibit mild sensory disturbances or sphincter dysfunction [1].

Magnetic resonance imaging findings are typically unremarkable, though occasional contrast enhancement of the cauda equina nerve roots may be observed. Cerebrospinal fluid (CSF) analysis is generally acellular but frequently reveals elevated protein levels. Nerve conduction studies (NCSs) usually demonstrate preserved motor and sensory conduction velocities, with intact sensory nerve action potentials (SNAPs). However, F-wave responses may be absent or prolonged. Electromyographic (EMG) evaluation often detects spontaneous activity, including fasciculations, fibrillations, and positive sharp waves. A key neurophysiological hallmark suggestive of radiation-induced neuropathy is the presence of myokymic discharges—characteristic spontaneous, rhythmic, and repetitive motor unit discharges that are infrequently encountered during needle EMG [1]. Notably, radiation-induced myokymia is reported to exhibit distinct morphological patterns, potentially aiding in differential diagnosis [2].

Osteoradionecrosis of the hip represents a rare but serious late complication of radiotherapy, one that is frequently under-recognized in its early stages by both clinicians and patients. Due to the limited number of published cases, a definitive consensus regarding its pathogenesis, early diagnostic markers, and optimal therapeutic strategies has yet to be established [3].

In this report, we describe a distinctive case of a 56-year-old female who developed a constellation of post-radiation complications—namely LMNS, myokymia, and osteoradionecrosis of the hip—nearly two decades after undergoing pelvic radiotherapy for cervical carcinoma. To the best of our knowledge, no previous reports have described this particular triad of neurological and tissue changes following radiation therapy. The case is contextualized within a broader systematic review of the literature to better elucidate the clinical spectrum and underlying mechanisms of such sequelae.

## 2. Case Presentation

A 56-year-old woman presented with a six-year history of progressive lower extremity weakness. She had been treated 19 years prior for FIGO II.B squamous cell carcinoma of the cervix with radical radio-chemotherapy (cisplatin 60 mg IV and 85 Gy pelvic radiation). The patient received a combination of external beam radiotherapy (EBRT) and intracavitary brachytherapy. EBRT was delivered with a pelvic box technique (total of 49.6 Gy in 1.6 Gy fractions, with an additional 4.8 Gy boost to pelvic nodes). This was followed by intracavitary brachytherapy according to the Manchester scheme, with a total dose of 40 Gy to Point A (a standard reference point for cervical brachytherapy, located near the uterine cervix). The cumulative dose to the cervix was approximately 85–90 Gy, while the surrounding pelvic tissues received 50–55 Gy. Field design included anterior–posterior/posterior–anterior (AP/PA) pelvic portals with paraaortic extension, followed by intracavitary uterine applicators.

In September 2022, she experienced three weeks of sharp right groin pain, progressive right leg weakness, and difficulty walking, without sphincteric or sensory disturbances. Clinical examination revealed proximal lower limb muscle atrophy and fasciculations, flaccid paraparesis with hyporeflexia (MRC hip flexors: left 1/5, right 2/5; knee flexors: left 3/5, right 4/5; dorsal flexion: 4+/5), and symmetrical brisk reflexes in the upper limbs. NCSs and EMG showed a chronic lower motor neuron lesion in cervical and lumbosacral body segments with signs of active denervation in at least two muscles of the lumbosacral body segment. SNAP values were normal, suggesting a preganglionic lesion.

Extensive laboratory and CSF analyses—including routine blood tests, arterial blood gas analysis, vitamin B12, folate, tumor markers, creatine kinase, myoglobin, thyroid hormones, serum protein electrophoresis, antiganglioside antibodies, and paraneoplastic antibodies—were unremarkable. Infectious causes were excluded. CSF analysis showed no albumin–cytologic dissociation, and cytology was negative for malignant cells. MRI of the brain and the cervical, thoracic, and lumbosacral spine revealed no structural changes indicative of myelopathy or significant foraminal narrowing; there was no contrast enhancement in the cauda equina. A diagnosis of motor neuron disease (pseudopolyneuritic variant or flail leg syndrome) was first made.

**Figure 1:**
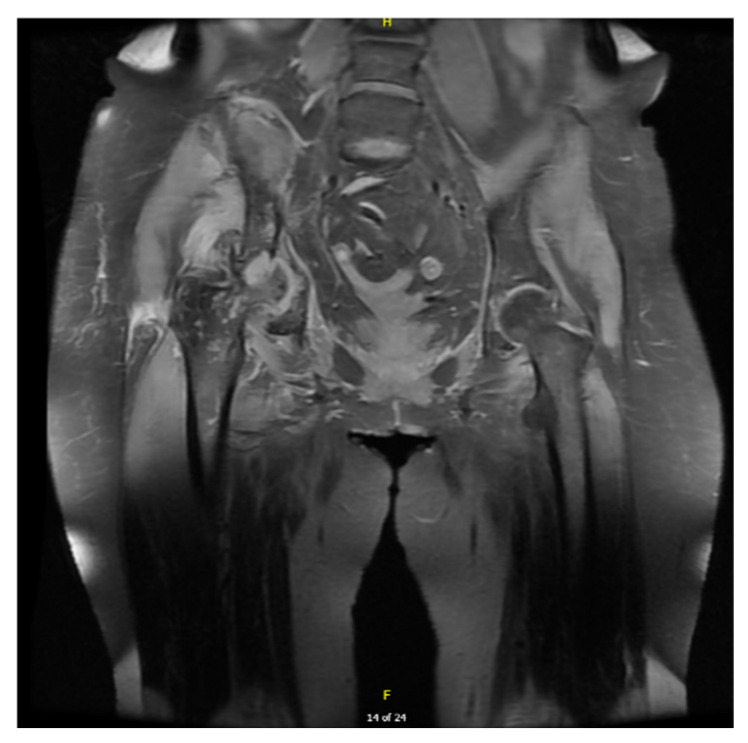
Coronal T1-weighted contrast-enhanced MRI of the pelvis demonstrating aseptic necrosis of the femoral head with surrounding bone marrow changes.

**Figure 2:**
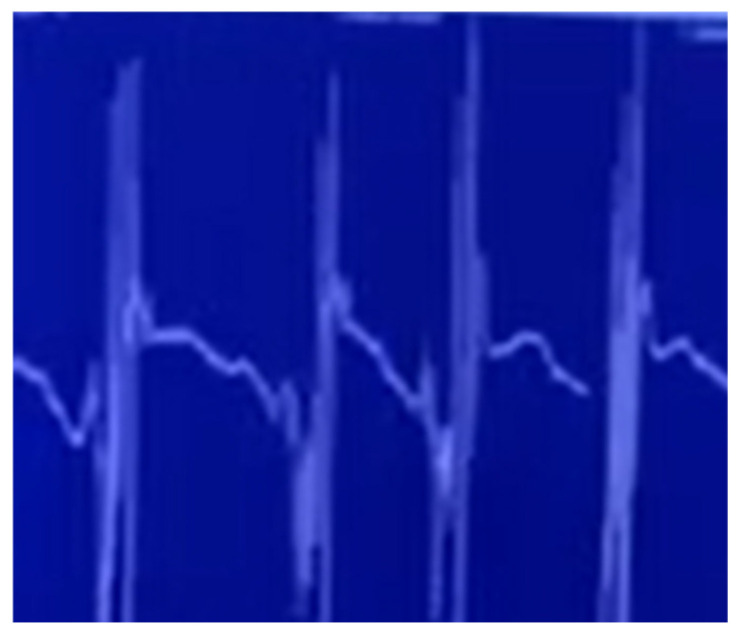
Needle electromyography (EMG) of the gluteus medius muscle demonstrates spontaneous myokymic discharges, characterized by rhythmic, grouped motor unit action potentials with regular inter-burst intervals. Such activity is considered a characteristic but infrequently reported feature of radiation-induced lower motor neuron injury.

Pelvic MRI with contrast revealed avascular necrosis with effusion in the right hip joint (Figure 1). Based on orthopedic consultation, total endoprosthesis (TEP) of the right hip was recommended and performed in January 2023 due to severe coxarthrosis. By February 2024, the patient exhibited worsening foot dorsiflexion (MRC 2/5) and could no longer walk independently. Follow-up NCSs and EMG performed in March 2024 indicated progressive lower motor neuron syndrome in the lumbosacral body segment with active denervation signs and identified myokymia in the gluteus medius muscle (Table 1, Table 2, Table 3 and Table 4, Figure 2; Video S1 is available in the Supplementary Materials). Based on these findings, the diagnosis of post-radiation LMNS was made. The patient has been monitored for more than four years now, with slowly progressive neurological symptoms but no upper motor neuron involvement.

## 3. Materials and Methods

This study employed a systematic review methodology to identify all published cases of post-radiation LMNS. The review protocol was registered in the PROSPERO database (CRD420251070308) prior to the initiation of the review process https://www.crd.york.ac.uk/PROSPERO/view/CRD420251070308 (accessed on 13 September 2025). The review was conducted following the PRISMA (Preferred Reporting Items for Systematic Reviews and Meta-Analyses) guidelines, with adaptations appropriate for rare case reports and small case series [4]. A completed PRISMA 2020 checklist is provided in the Supplementary Table S1.

A systematic literature search was conducted in PubMed, Web of Science, and Google Scholar (last search: 13 September 2025) using predefined Boolean strings with controlled vocabulary (MeSH/EMTREE) and synonyms (full strategies are provided in Supplementary Table S2). Searches were not limited by year; animal-only records were excluded.

The inclusion criteria were (1) case reports or case series describing post-radiation LMNS; (2) sufficient clinical, radiological, or electrophysiological details to confirm the diagnosis; (3) publication in peer-reviewed journals. Exclusion criteria were (1) reviews, editorials, or theoretical articles without individual patient data and (2) duplicate reports or overlapping data from the same patient cohort. Manual cross-referencing of bibliographies from identified articles was also performed to capture additional relevant cases. A total of 58 cases, including our own, were included in the final analysis and are presented in Supplementary Table S3 [5,6,7,8,9,10,11,12,13,14,15,16,17,18,19,20,21,22,23,24,25,26,27,28].

Two independent reviewers (T.P. and M.M.) screened all articles for eligibility. Disagreements were resolved through consensus or consultation with a third reviewer (S.G.). The quality of included case reports and case series was assessed using the Joanna Briggs Institute (JBI) Critical Appraisal Checklist for Case Reports [29].

The inclusion criteria for LMNS were based on clinical and electrophysiological features consistent with anterior horn cell involvement following radiotherapy. Patients had to present with progressive lower motor neuron weakness and atrophy, without sensory or sphincter disturbance as the main feature. Mild sensory complaints were allowed, provided that SNAPs were preserved on NCSs. EMG had to demonstrate evidence of acute and/or chronic denervation (fibrillations, positive sharp waves, neurogenic motor unit potentials). MRI or CT imaging was required to exclude tumor recurrence or compressive lesions. However, where NCSs/EMG and/or imaging were not performed or not reported, cases were included if the clinical presentation and available investigations were consistent with LMNS and no suspicion of recurrence was raised by the authors.

For all included patients, radiotherapy modality was categorized as external beam radiotherapy (EBRT). No cases of brachytherapy were reported. All radiation doses are reported in Gray (Gy), in accordance with SI units. Values originally recorded in centigray (cGy) were converted (100 cGy = 1 Gy).

Descriptive and inferential statistics were used to analyze the clinical, electrophysiological, and radiologic characteristics of patients with LMNS. Categorical variables were compared using Fisher’s exact test for 2 × 2 tables, and the Chi-squared (χ^2^) test for larger contingency tables when cell sizes permitted. For continuous variables, the Mann–Whitney U test was applied due to non-normal distribution and small subgroup sizes. Statistical significance was set at *p* ≤ 0.05. All analyses were performed using “scipy.stats” Python library (version 1.11.3, running on Python 3.11.5.). No imputation was performed for missing values.

## 4. Results

### 4.1. Study Selection

The database searches yielded 530 records (PubMed: 149; Google Scholar: 244; Web of Science: 137). After screening the titles and abstracts, 37 full-text articles were retrieved and assessed for eligibility. Five articles were excluded after full-text review [30,31,32,33,34]. In total, 32 studies were included in the qualitative synthesis, reporting 57 unique cases of post-radiation LMNS. With the addition of our present case, the final analysis comprised 58 cases. The study selection process is summarized in the PRISMA 2020 flow diagram (Supplementary Figure S1).

### 4.2. Quality Assessment of Included Case Reports

The methodological quality of the included case reports and case series was assessed using a modified Joanna Briggs Institute (JBI) Critical Appraisal Checklist for Case Reports. Out of 57 identified cases, none achieved a perfect score across all eight evaluated domains. Twelve cases (21.1%) were rated as high-quality, most often meeting all JBI criteria except for minor omissions. The majority of reports (75.4%) were of moderate quality, typically lacking a detailed description of treatment or adverse events. Two cases (3.5%) were considered low-quality due to incomplete documentation of clinical presentation or diagnostic workup. Despite these limitations, the overall methodological quality was deemed sufficient to support the subsequent analysis. Detailed results of the quality assessment are presented in Supplementary Table S4.

### 4.3. Clinical, Demographic, and Radiological Characteristics of the Cohort

**Figure 3:**
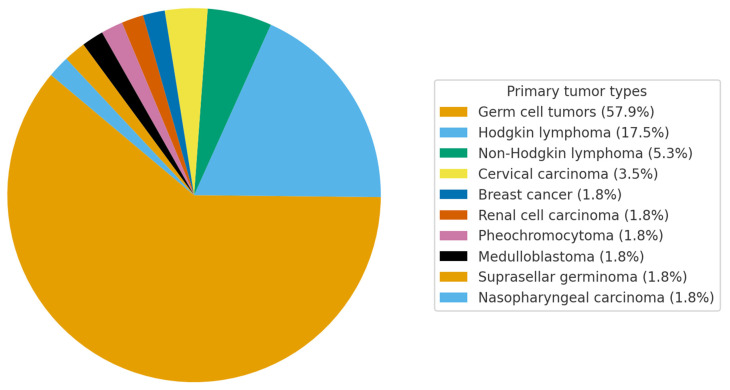
Graphical representation of the proportion of each malignancy in post-radiation LMNS.

In a cohort of patients with post-radiation LMNS, the mean age at symptom onset was 40.5 ± 13 years, with radiotherapy administered at a mean age of 30.3 ± 12.5 years. The latency period between radiation exposure and the emergence of neurological symptoms averaged 10.2 ± 8.7 years. All patients in the cohort had received EBRT (photons or electrons), most commonly cobalt-60 or linac photons to the paraaortic/pelvic fields. No brachytherapy cases were identified. The mean cumulative radiation dose was 49.0 ± 14.0 Gy. The majority of patients were male (77.2%). The most frequent primary malignancies were germ cell tumors (57.9%) and Hodgkin lymphoma (17.5%), while other tumor types—including cervical, breast, renal, pheochromocytoma, medulloblastoma, suprasellar germinoma, and nasopharyngeal carcinoma—each accounted for <5% of cases (Figure 3). Sensory and/or sphincter involvement was noted in 23.1% of individuals. The distribution of motor weakness was predominantly distal in 45.5%, both proximal and distal in 42.4%, and purely proximal in 9.1%. Asymmetry of weakness was observed in 50% of patients. Myokymic discharges were detected in only 10.3% of all cases (12.8% of those with reported EMG data). Treatment was supportive in all reported cases, except for one patient [8] who received corticosteroids and warfarin with partial improvement. No other disease-directed interventions were reported.

No statistically significant differences were identified in clinical or radiological features among patients with varying oncological diagnoses. When stratified by tumor subgroup, myokymic discharges were observed in 2/21 (9.5%) patients with germ cell tumors, 0/11 (0%) with lymphoma, and 4/15 (26.7%) with other malignancies. No statistically significant differences were detected (Germ cell vs. Lymphoma: Fisher’s exact *p* = 0.53, OR = ∞, 95% CI 0.10–56.1; Germ cell vs. Other: *p* = 0.21, OR = 0.29, 95% CI 0.045–1.85; Lymphoma vs. Other: *p* = 0.11, OR = 0.0, 95% CI 0.0059–2.66). Sensory and sphincter involvement was present in 12/52 (23.1%) evaluable cases; however, the distribution across tumor groups was too sparse to allow for statistical testing. Similarly, SNAPs were preserved in 48/51 (94.1%), and gadolinium-enhancing cauda equina lesions were noted in 8/35 (22.9%), without clear differences between tumor subgroups. Taken together, these analyses suggest that subgroup comparisons are underpowered and should be interpreted with caution.

The clinical and demographic characteristics of the cohort are presented in Supplementary Table S3 [5,6,7,8,9,10,11,12,13,14,15,16,17,18,19,20,21,22,23].

## 5. Discussion

Post-radiation LMNS represents a rare yet clinically consequential delayed complication of radiation therapy. Initially described in 1947, it remains an uncommon manifestation, particularly in patients treated for cervical cancer [35]. The syndrome is typified by insidiously progressive motor deficits, including muscle weakness, atrophy, and fasciculations, occurring without associated sensory impairment. Its protracted latency period following radiation exposure poses diagnostic challenges and may delay appropriate management.

Our case is particularly distinctive, presenting with both myokymia and avascular necrosis of the hip—an uncommon combination not previously reported in the literature. The extended latency observed in our patient also contrasts with the shorter intervals described by Umeda et al. [36]. Importantly, no structural abnormalities of the spinal cord or cauda equina were identified, in contrast to prior cases where such changes were observed [6,8,11,36]. This variability underscores the need for a comprehensive diagnostic framework integrating advanced imaging, detailed clinical assessment, and electrophysiological evaluation. At presentation, the patient was initially suspected of having a motor neuron disease (pseudopolyneuritic variant or flail leg syndrome) due to slow-progressing, asymmetric lower limb weakness and wasting. However, several features supported an alternative diagnosis of post-radiation LMNS: a history of high-dose pelvic radiotherapy as a recognized risk factor; the presence of myokymia on EMG, which is characteristic of radiation-induced anterior horn cell injury but rare in primary motor neuron disease; a slowly progressive course confined to the irradiated lumbosacral segments, without spread to the bulbar, cervical, or thoracic regions and without development of upper motor neuron signs; and the occurrence of aseptic femoral head necrosis, a well-established late complication of pelvic irradiation. Taken together, these findings strongly support the diagnosis of post-radiation LMNS rather than a primary motor neuron disease.

In a systematic review, we evaluated 58 documented cases of post-radiation LMNS, with particular emphasis on clinical presentation, electrophysiological characteristics, and neuroimaging findings. The mean age at symptom onset was 40.5 years, and the average latency between radiation exposure and the development of neurological symptoms was 10.2 years. These observations are consistent with the prior literature, which highlights the heterogeneous and often prolonged latency associated with post-radiation LMNS. This variability reinforces the critical importance of extended surveillance in individuals who have received radiation therapy [5,6,7,8,9,10,11,12,13,14,15,16,17,18,19,20,21,22,23].

The malignancies most frequently associated with post-radiation LMNS were testicular germ cell tumors and Hodgkin’s lymphoma. Testicular cancer, in particular, demonstrated a higher incidence of LMNS, with a substantial proportion of cases exhibiting bilateral, predominantly distal motor weakness [7,8,9,10,11,13,14,15,18,19,20,23].

The reported radiation doses varied considerably, ranging from 25 to 90 Gy (mean 49.0 ± 14.0 Gy), without evidence of a clear dose–response relationship. This lack of correlation suggests that, although higher cumulative doses may contribute to risk, additional factors—such as irradiated volume, field localization, and individual susceptibility—likely play a crucial role in the pathogenesis of LMNS. Almost all cases were treated with EBRT, which facilitates uniform interpretation but precludes meaningful comparison with other modalities such as brachytherapy or combined approaches.

A comparative analysis of clinical and radiological characteristics across malignancy subgroups did not reveal any statistically significant differences. Small sample sizes and sparse data limited the power of subgroup analyses, and observed numerical trends should be interpreted cautiously. A tendency toward longer latency intervals was observed in patients with lymphoma and central nervous system tumors, although this did not reach statistical significance. Distal motor involvement appeared numerically more frequently in Hodgkin’s lymphoma and seminoma, while sensory and sphincter disturbances were most often reported in testicular germ cell tumors. Myokymic discharges and gadolinium-enhancing cauda equina lesions on MRI were rare across all tumor types, with no clear clustering by subgroup. These findings suggest that the clinical expression of post-radiation LMNS is broadly similar across oncological backgrounds and that previously suggested subgroup differences may largely reflect reporting variability rather than distinct pathophysiological patterns.

Electrophysiological assessments consistently demonstrated findings indicative of lower motor neuron involvement, with neurogenic changes identified in more than 90% of cases. The frequent preservation of SNAPs and absence of sensory deficits further substantiated the diagnosis of LMNS, effectively distinguishing it from cauda equina syndrome or postganglionic lesions. A noteworthy electrophysiological feature was the presence of myokymia, documented in 6/58 cases (10.3%), or 12.8% when restricted to patients with reported EMG data. Although considered a hallmark of radiation-induced neuropathy, myokymia was less frequent than expected. Several explanations may account for this discrepancy. First, myokymia is often subtle, intermittent, and easily overlooked, particularly in early reports with limited EMG sensitivity. Second, differences in the muscles examined and the duration of recordings likely contributed to underreporting. Third, radiotherapy parameters—such as field configuration, fractionation, or cumulative dose to the plexus or anterior horn cells—may influence its occurrence, although current evidence is insufficient to establish a causal link. Finally, some cases labeled as LMNS may overlap with radiation-induced plexopathy, where myokymia is more frequently encountered. Thus, while uncommon in published series, the presence of myokymia in our index patient underscores its potential diagnostic value and further supports a radiation-induced pathogenesis [5,23].

Neuroimaging revealed gadolinium enhancement of the cauda equina in 8/35 evaluable cases (22.9%), a finding of clinical significance given its potential to mimic alternative pathologies and thereby obscure accurate diagnosis. The diagnosis of LMNS remains challenging because of its clinical overlap with other radiation-induced entities such as lumbosacral plexopathy, radiculopathy, or cauda equina syndrome. While strict inclusion criteria were applied in our review, a subset of cases demonstrated mild sensory symptoms, occasional bladder dysfunction, or gadolinium enhancement of lumbosacral roots on MRI. These features may point toward a pathophysiological continuum between anterior horn cell involvement and radiation-induced plexo-radiculopathies. To accommodate this overlap, we included cases with minimal sensory changes provided that SNAPs were preserved, which is considered a key electrophysiological discriminator. This approach recognizes the spectrum of radiation-induced lower motor neuron injury while minimizing misclassification with predominantly sensory or compressive syndromes.

Therapeutic interventions were largely absent across the reported cases. Only one patient [8] received corticosteroids and warfarin, with a reported subjective clinical improvement. However, this improvement was not objectively confirmed by electrophysiological or imaging follow-up, and no other disease-directed treatments were described in the literature. Taken together, this highlights the lack of effective and validated therapeutic options for LMNS, emphasizing the need for further investigation into potential neuroprotective strategies.

This study has several limitations inherent to its design and data sources. First, the systematic review included only published case reports and case series, which may be subject to publication bias, favouring the reporting of more severe or atypical cases. Second, the data extracted from these reports were often incomplete or heterogeneously presented, limiting the uniformity and depth of the analysis. For example, electrophysiological details, latency periods, and radiation dosages were not consistently reported, and information on MRI findings was frequently absent. Third, heterogeneity in radiotherapy techniques—spanning Co-60, conventional X-rays, and electron beams with variable fields and fractionation—further complicates dose–response assessment. Fourth, many older cases predated the widespread availability of MRI and modern EMG standards, which may have contributed to systematic underreporting of features such as gadolinium enhancement or myokymia. Fifth, the sample size for subgroup comparisons—particularly for rare findings such as myokymia—was small, reducing statistical power and generalizability. Sixth, the methodological quality of the included reports was variable; only ~21% fulfilled all JBI criteria, and most lacked detailed descriptions of treatment, which limits their interpretability. Finally, although we searched multiple databases, the lack of Embase access may have introduced a search bias, and due to the retrospective nature of the study and its reliance on secondary data, causality between radiation exposure and neurological outcomes cannot be definitively established. Prospective, multicenter studies with standardized reporting are needed to validate and expand upon these findings.

In summary, the present report adds novelty in several respects. First, it provides one of the longest documented follow-ups of post-radiation LMNS, demonstrating a slowly progressive but highly localized course over many years without generalization to other body regions. Second, it highlights the presence of myokymic discharges, an electrophysiological feature rarely reported in LMNS, thereby expanding the neurophysiological spectrum of this entity. Third, the concomitant development of avascular femoral head necrosis further supports a radiation-induced etiology and illustrates how different late complications of pelvic irradiation can coexist in the same patient. Finally, by combining a detailed single-case description with a systematic review of all available cases, this work provides the most comprehensive synthesis to date of the clinical, electrophysiological, and radiological characteristics of LMNS. Taken together, these aspects underline the contribution of our study in clarifying the phenotype and natural history of this rare radiation-induced complication.

## 6. Conclusions

In conclusion, post-radiation LMNS remains a challenging condition with a diverse clinical presentation. The data from this review highlight the importance of considering LMNS in patients with a history of radiation therapy who present with motor symptoms. Early recognition and appropriate diagnostic evaluation, including electromyography and advanced imaging, are crucial for differentiating LMNS from other neurodegenerative conditions. As the field progresses, ongoing research and case documentation will be essential for improving our understanding of this rare syndrome and optimizing patient management.

## Figures and Tables

**Table 1:** Presents the results of motor nerve conduction measurements.

Nerve/Recording Site/Stimulation Site	Latency (ms)/(Normal Values)	Amplitude (mV)/(Normal Values)	Duration (ms)/(Normal Values)	Velocity (m/s)/(Normal Values)
Left Median-APB				
Wrist	3.59 (≤4.3)	16.1 (≥10)	6.25 (≤10)	
Elbow	7.08	15.9	6.82	65.9 (≥50)
Left Ulnar-ADM				
Wrist	2.71 (≤3.3)	20.0 (≥10)	6.09 (≤10)	
Bellow Elbow	5.52	19.4	5.57	71.1 (≥50)
Left Peroneal-EDB				
Ankle	4.58 (≤6.0)	**1.5** (≥2)	4.38 (≤10)	
Bellow Fibular Head	11.46	**1.5**	4.95	**40.7** (≥43)
Right Peroneal				
Ankle	2.71	12.6 (≥2)	5.42	
Bellow Fibular Head	9.11	10.4	5.73	45.3
Left Tibial-AH				
Ankle	4.90 (≤6.0)	**0.8** (≥2)	9.01 (≤10)	
Knee	14.22	0.7	9.53	**40.8** (≥43)
Right Tibial-AH				
Ankle	2.92	13.0 (≥ 2)	6.20	

APB: abductor pollicis brevis; ADM: abductor digiti minimi; EDB: extensor digitorum brevis; AH: abductor hallucis. Abnormal values are indicated in bold.

**Table 2:** Presents the results of sensory nerve conduction measurements.

Nerve/Stimulation Site	Recording Site	Latency (ms)/(Normal Values)	Amplitude (μV)/(Normal Values)	Distance (cm)/(Normal Values)	Velocity (m/s)/(Normal Values)
Left Median					
Wrist	II finger	2.45 (≤3.0)	17.1(≥10)	14	57.2 (≥50)
Left Ulnar					
Wrist	V finger	2.19 (≤3.0)	16.6 (≥10)	14	64.0 (≥50)
Left Sural					
Calf	Ankle	2.45	11.6 (≥10)	14	57.2 (≥50)

**Table 3:** Presents the results of the minimal F wave latencies.

Nerve/Recording Site	Minimal F Wave Latency (ms)/(Normal Values)
Left ulnar—ADM	23.6 (≤33)
Left peroneal—EDB	**62.2** (≤55)
Left tibial—AH	**Absent**
Right peroneal—EDB	**58.6** (≤55)
Right tibial—AH	**59.1** (≤55)

ADM: abductor digiti minimi; EDB: extensor digitorum brevis; AH: abductor hallucis. Abnormal values are indicated in bold.

**Table 4:** Needle EMG.

Needle EMG	Spontaneous Activity			MUAP					
Muscle	Fib.	PSW	Fasciculations	Amplitude (mV)	Duration	Polyphasicity	Pattern Reduction	CRD	Other
Left tibialis anterior	**2+**	**2+**	No	**7**	**1+**	**1+**	**3+**	No	No
Left gastrocnemius (Medial head)	**3+**	**3+**	No	**10**	**1+**	**1+**	**3+**	No	No
Left vastus lateralis	**3+**	**3+**	No	**8**	**1+**	N	**2–3+**	No	No
Right tibialis Anterior	No	No	No	**5**	**1+**	**1+**	**3+**	No	Prolonged insertional activity
Left first dorsal interosseous	No	No	No	**5**	**1+**	**1+**	**N/1+**	No	No
Left deltoid	No	No	No	2–5	**1+**	**2+**	**N/1+**	No	No
Left masseter	No	No	No	2	N	N	N	No	No
Left gluteus medius	No	No	No	4	**1+**	**2+**	**2+**	No	Prolonged insertional activity, myokymia

Fib.: fibrillation potentials; PSWs: positive sharp waves; CRDs: chronic repetitive discharges. Abnormal values are indicated in bold.

## Data Availability

The data presented in this study are available on request from the corresponding author. Requests to access the datasets should be directed to Timotej Petrijan at timotej.petrijan@gmail.com.

## Supplementary Materials

The following supporting information can be downloaded at https://www.mdpi.com/article/10.3390/jcm14196955/s1. Figure S1: PRISMA 2020 Flow Diagram; Table S1: PRISMA 2020 checklist; Table S2: Search Strategies; Table S3: Demographic, clinical, electrophysiological and radiologic characteris-tics in postradiation LMNS; Table S4: JBI Quality Assessment; Video S1: Myokymia.

## Abbreviations

The following abbreviations are used in this manuscript:

LMNS	Lower Motor Neuron Syndrome
CSF	Cerebrospinal Fluid
NCSs	Nerve Conduction Studies
SNAPs	Sensory Nerve Action Potentials
EMG	Electromyography
EBRT	External beam radiotherapy
PRISMA	Preferred Reporting Items for Systematic Reviews and Meta-Analyses
JBI	Joanna Briggs Institute

## References

[1] Kosmin M., Rees J. (2022). Radiation and the Nervous System. Pract. Neurol..

[2] Oishi T., Ryan C.S., Vazquez Do Campo R., Laughlin R.S., Rubin D.I. (2021). Quantitative Analysis of Myokymic Discharges in Radiation versus Nonradiation Cases. Muscle Nerve.

[3] Xu S.H., Tang J.S., Shen X.Y., Niu Z.X., Xiao J.L. (2022). Osteoradionecrosis of the Hip, a Troublesome Complication of Radiation Therapy: Case Series and Systematic Review. Front. Med..

[4] Page M.J., McKenzie J.E., Bossuyt P.M., Boutron I., Hoffmann T.C., Mulrow C.D., Shamseer L., Tetzlaff J.M., Akl E.A., Brennan S.E. (2021). The PRISMA 2020 Statement: An Updated Guideline for Reporting Systematic Reviews. BMJ.

[5] Matsuda N., Kobayashi S., Matsumoto H., Machii M., Soeda T., Ugawa Y. (2015). Cauda Equina Involvement in Post-Radiation Lower Motor Neuron Syndrome. Intern. Med..

[6] Hsia A.W., Katz J.S., Hancock S.L., Peterson K. (2003). Post-Irradiation Polyradiculopathy Mimics Leptomeningeal Tumor on MRI. Neurology.

[7] Van Der Sluis R.W., Wolfe G.I., Nations S.P., Bryan W.W., Krampitz D.E., Kissel J.T., Barohn R.J. (2000). Post-Radiation Lower Motor Neuron Syndrome. J. Clin. Neuromuscul. Dis..

[8] Anezaki T., Harada T., Kawachi I., Sanpei K., Soma Y., Tsuji S. (1999). A case of post-irradiation lumbosacral radiculopathy successfully treated with corticosteroid and warfarin. Rinsho Shinkeigaku.

[9] Wohlgemuth W.A., Rottach K., Jaenke G., Stöhr M. (1998). Radiogenic Amyotrophy. Cauda Equina Lesion as a Late Radiation Sequela. Nervenarzt.

[10] Tallaksen C.M.E., Jetne V., Fosså S. (2009). Acta Oncologica Postradiation Lower Motor Neuron Syndrome: A Case Report and Brief Literature Review. Acta Oncol..

[11] Bowen J., Gregory R., Squier M., Donaghy M. (1996). The Post-Irradiation Lower Motor Neuron Syndrome Neuronopathy or Radiculopathy?. Brain.

[12] Grunewald R.A., Chroni E., Panayiotopoulos C.P., Enevoldson T.P. (1992). Late Onset Radiation-Induced Motor Neuron Syndrome. J. Neurol. Neurosurg. Psychiatry.

[13] Jackson M. (1992). Post Radiation Monomelic Amyotrophy. J. Neurol. Neurosurg. Psychiatry.

[14] Lamy C., Mas J.L., Varet B., Ziegler M., de Recondo J. (1991). Postradiation Lower Motor Neuron Syndrome Presenting as Monomelic Amyotrophy. J. Neurol. Neurosurg. Psychiatry.

[15] Feistner H., Weissenborn K., Münte T.F., Heinze H.-J., Malin J.P. (1989). Post-Irradiation Lesions of the Caudal Roots. Acta Neurol. Scand..

[16] Gállego J., Delgado G., Tuñón T., Villanueva J.A. (1986). Delayed Postirradiation Lower Motor Neuron Syndrome. Ann. Neurol..

[17] De Carolis P., Montagna P., Cipulli M., Baldrati A., D’Alessandro R., Sacquegna T. (1986). Isolated Lower Motoneuron Involvement Following Radiotherapy. J. Neurol. Neurosurg. Psychiatry.

[18] Lagueny A., Aupy M., Aupy P., Ferrer X., Henry P., Julien J. (1985). Post-radiotherapy anterior horn cell syndrome. Rev. Neurol..

[19] Horowitz S.L., Stewart J.D. (1983). Lower Motor Neuron Syndrome Following Radiotherapy. Can. J. Neurol. Sci..

[20] Schiødt A.V., Kristensen O. (1978). Neurologic Complications after Irradiation of Malignant Tumors of the Testis. Acta Radiol. Oncol. Radiat. Phys. Biol..

[21] Kristensen O., Melgård B., Schiødt A.V. (1977). Radiation Myelopathy of the Lumbo-Sacral Spinal Cord. Acta Neurol. Scand..

[22] Sadowsky C.H., Sachs E., Ochoa J. (1976). Postradiation Motor Neuron Syndrome. Arch. Neurol..

[23] Abraham A., Drory V.E. (2013). Postradiation Lower Motor Neuron Syndrome: Case Series and Literature Review. J. Neurol..

[24] Delanian S., Pradat P.F. (2010). Posteriori Conformal Radiotherapy Using Three-Dimensional Dosimetric Reconstitution in a Survivor of Adult-Onset Hodgkin’s Disease for Definitive Diagnosis of Lower Motor Neuron Disease. J. Clin. Oncol..

[25] Ésik O., Lengyel Z., Sáfrány G., Vönöczky K., Ágoston P., Székely J., Lengyel E., Márián T., Trón L., Bodrogi I. (2002). A PET Study on the Characterization of Partially Reversible Radiogenic Lower Motor Neurone Disease. Spinal Cord..

[26] De Greve J.L.P., Bruyland M., De Keyser J., Storme G., Ebinger G. (1984). Lower Motor Neuron Disease in a Patient with Hodgkin’s Disease Treated with Radiotherapy. Clin. Neurol. Neurosurg..

[27] Shapiro B.E., Rordorf G., Schwamm L., Preston D.C. (1996). Delayed Radiation-Induced Bulbar Palsy. Neurology.

[28] Giray E., Karayigit M., Senocak K.C., Illeez O.G., Ozkan F.U., Aktas I., Gozke E. (2023). Delayed Radiation-Induced Motor Neuron Syndrome: A Case Report. J. Back. Musculoskelet. Rehabil..

[29] Gagnier J.J., Kienle G., Altman D.G., Moher D., Sox H., Riley D., Allaire A., Aronson J., Carpenter J., Gagnier J. (2013). The CARE Guidelines: Consensus-Based Clinical Case Reporting Guideline Development. BMJ Case Rep..

[30] Knap M.M., Bentzen S.M., Overgaard J. (2007). Late Neurological Complications after Irradiation of Malignant Tumors of the Testis. Acta Oncol..

[31] Raheem O.A., Hickey D.P. (2011). Postirradiation Lumbosacral Radiculopathy Following Seminoma Treatment Presenting as Flaccid Neuropathic Bladder: A Case Report. J. Med. Case Rep..

[32] Tan S.V., Pye I.F. (1991). Postradiation Motor Neuron Syndrome of the Upper Cervical Region—A Manifestation of the Combined Effect of Cranial Irradiation and Intrathecal Chemotherapy?. J. Neurol. Neurosurg. Psychiatry.

[33] Memon A.B., Playfoot K.A. (2017). Radiation—induced Tongue Myokymia with Hypoglossal Nerve Damage, Mimicker of Motor Neuron Disease. Clin. Case Rep..

[34] Bhatia S., Miller R.C., Lachance D.L. (2006). Neck Extensor Muscle Weakness (Dropped Head Syndrome) Following Radiotherapy. Radiol. Oncol..

[35] Greenfield M.M., Stark F.M. (1948). Post-Irradiation Neuropathy. Am. J. Roentgenol. Radium Ther..

[36] Umeda M., Naruse S., Ito A., Fujita N. (2005). Gandolinium Enhancement of the Anterior Portion of the Lumbosacral Roots in a Case of Post-Irradiation Lumbosacral Radiculopathy. Rinsho Shinkeigaku.

