# Supplementary material for: A Puzzling Pair: Flail Leg Syndrome with Myokymia and Avascular Hip Necrosis—Case Study and Systematic Literature Review
Source: J Clin Med. 2025 Oct 1;14(19):6955. doi: 10.3390/jcm14196955 (PMC12524704; doi:10.3390/jcm14196955)
Supplement: Supplementary file 1 [file jcm-14-06955-s001.zip › Supplementary Table S2_Search_Strategies_final.pdf]

## Supplementary Table S2: Search Strategies in PubMed, Web of Science, Google Scholar

This supplementary file provides the full search strategies applied in each database, together with the date of the last search (13 September, 2025). Searches were not limited by year; animal-only records were excluded.

| Database | Search string | Date searched |
|---|---|---|
| PubMed | ("postradiation"[All Fields] OR "post-irradiation"[All Fields] OR "radiation-induced"[All Fields] OR "irradiation"[All Fields]) AND ("lower motor neuron"[All Fields] OR "anterior horn cell"[All Fields] OR "lumbosacral radiculopathy"[All Fields] OR "cauda equina"[All Fields] OR "radiogenic amyotrophy"[All Fields] OR "myokymia"[All Fields]) NOT (animals[mh] NOT humans[mh]) | 13 September 2025 |
| Web of Science | TS=(postradiation OR "post-irradiation" OR "radiation-induced") AND<br>TS=("lower motor neuron" OR "anterior horn cell" OR "lumbosacral radiculopathy"<br>OR "cauda equina" OR "radiogenic amyotrophy" OR myokymia) | 13 September 2025 |
| Google Scholar | Multiple queries:<br>- "postradiation" "lower motor neuron"<br>- "post-irradiation" "lower motor neuron"<br>- "radiogenic amyotrophy" cauda equina<br>- "radiation-induced" myokymia lumbosacral | 13 September 2025 |
