# Supplementary figures and images for: A Puzzling Pair: Flail Leg Syndrome with Myokymia and Avascular Hip Necrosis—Case Study and Systematic Literature Review
Source: J Clin Med. 2025 Oct 1;14(19):6955. doi: 10.3390/jcm14196955 (PMC12524704; doi:10.3390/jcm14196955)

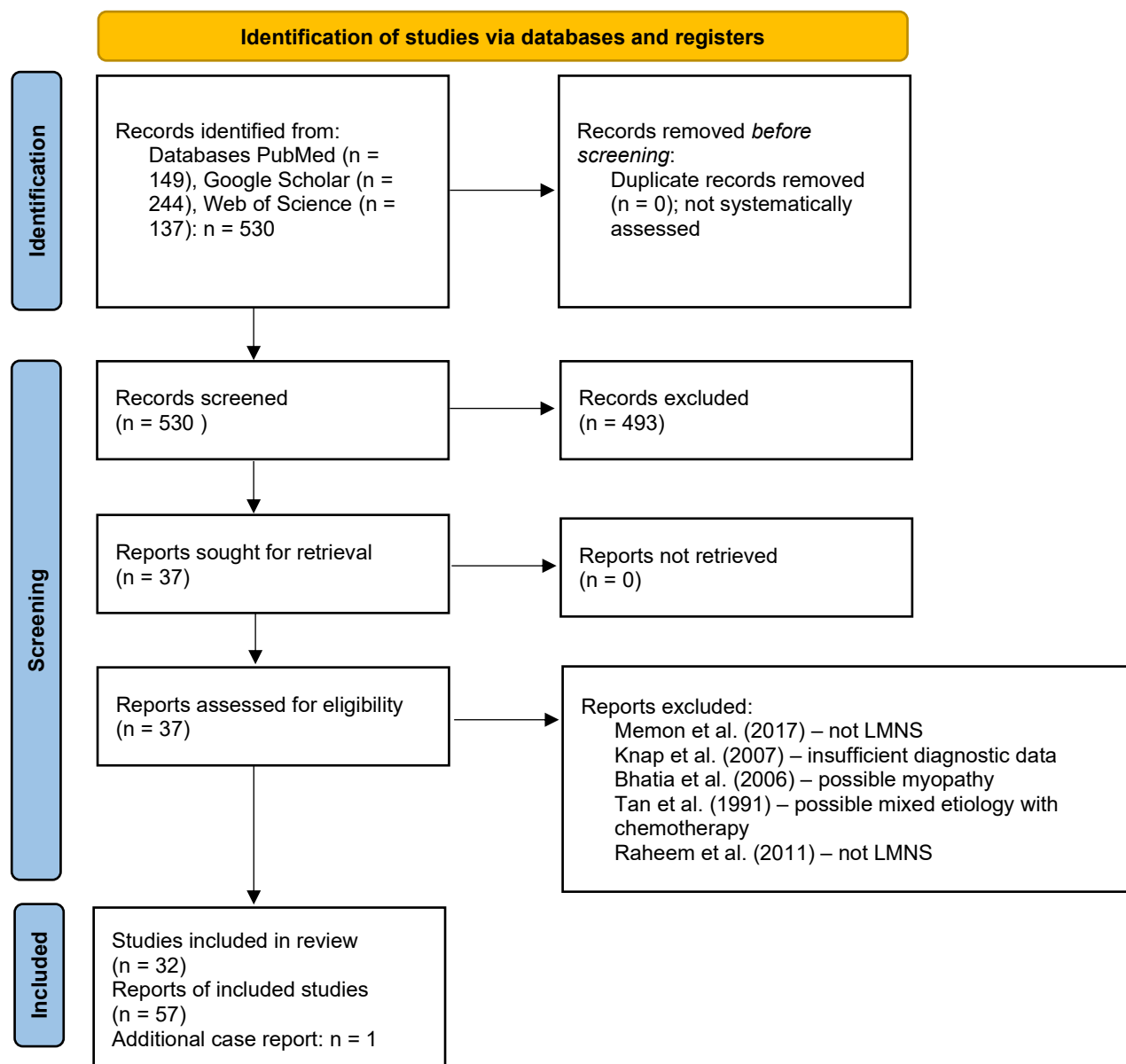

Supplement: Supplementary file 1 [file jcm-14-06955-s001.zip › Supplementary Figure S1 PRISMA_2020_flow_diagram_final.pdf]
